# JAK2/STAT3 Signaling in Myeloid Cells Contributes to Obesity-Induced Inflammation and Insulin Resistance

**DOI:** 10.3390/cells14151194

**Published:** 2025-08-02

**Authors:** Chunyan Zhang, Jieun Song, Wang Zhang, Rui Huang, Yi-Jia Li, Zhifang Zhang, Hong Xin, Qianqian Zhao, Wenzhao Li, Saul J. Priceman, Jiehui Deng, Yong Liu, David Ann, Victoria Seewaldt, Hua Yu

**Affiliations:** 1 Department of Immuno-Oncology, Beckman Research Institute at City of Hope National Medical Center, Duarte, CA 91010, USA; jisong316@gmail.com (J.S.); kenneth.zhang@legendbiotech.cn (W.Z.); ruhuang@coh.org (R.H.); yili@coh.org (Y.-J.L.); hoxin@ucsd.edu (H.X.); zhaoqianqian1116@gmail.com (Q.Z.); wenzhao.lee@gmail.com (W.L.); priceman@usc.edu (S.J.P.); jiehui.deng@nyulangone.org (J.D.); liu6yong@gmail.com (Y.L.); 2 Department of Surgery, City of Hope National Medical Center, Beckman Research Institute at City of Hope National Medical Center, Duarte, CA 91010, USA; zhzhang@coh.org; 3 Department of Medicine, KSOM/Norris Center for Cancer Cellular Immunotherapy Research, Keck School of Medicine of USC, Los Angeles, CA 90033, USA; 4 Department of Diabetes and Metabolism, Beckman Research Institute at City of Hope National Medical Center, Duarte, CA 91010, USA; dann@coh.org; 5 Department of Population Sciences, Beckman Research Institute at City of Hope National Medical Center, Duarte, CA 91010, USA; vseewaldt@coh.org

**Keywords:** JAK2/STAT3, obesity, insulin resistance, inflammation

## Abstract

Adipose tissue inflammation contributes to obesity-induced insulin resistance. However, increasing evidence shows that high BMI (obesity) is not an accurate predictor of poor metabolic health in individuals. The molecular mechanisms regulating the metabolically activated M1 macrophage phenotype in the adipose tissues leading to insulin resistance remain largely unknown. Although the Janus Kinase (Jak)/signal transducer and activator of transcription 3 (Stat3) signaling in myeloid cells are known to promote the M2 phenotype in tumors, we demonstrate here that the Jak2/Stat3 pathway amplifies M1-mediated adipose tissue inflammation and insulin resistance under metabolic challenges. Ablating Jak2 in the myeloid compartment reduces insulin resistance in obese mice, which is associated with a decrease in infiltration of adipose tissue macrophages (ATMs). We show that the adoptive transfer of Jak2-deficient myeloid cells improves insulin sensitivity in obese mice. Furthermore, the protection of obese mice with myeloid-specific Stat3 deficiency against insulin resistance is also associated with reduced tissue infiltration by macrophages. Jak2/Stat3 in the macrophage is required for the production of pro-inflammatory cytokines that promote M1 macrophage polarization in the adipose tissues of obese mice. Moreover, free fatty acids (FFAs) activate Stat3 in macrophages, leading to the induction of M1 cytokines. Silencing the myeloid cell Stat3 with an in vivo siRNA targeted delivery approach reduces metabolically activated pro-inflammatory ATMs, thereby alleviating obesity-induced insulin resistance. These results demonstrate Jak2/Stat3 in myeloid cells is required for obesity-induced insulin resistance and inflammation. Moreover, targeting Stat3 in myeloid cells may be a novel approach to ameliorate obesity-induced insulin resistance.

## 1. Introduction

Obese individuals often develop resistance to insulin, characterized by the impaired ability of insulin to regulate glucose homeostasis [1,2,3]. However, a high BMI/being overweight itself is not a predictor of insulin resistance. Therefore, it is critical to understand the molecular mechanisms by which increased adipose mass in obese individuals predisposes them to systemic insulin resistance. Of interest, obesity is characterized by an increased accumulation of immune cells and the resultant inflammation in the adipose tissues [4,5,6,7,8,9,10]. A causative role of immune cells in promoting various diseases has been demonstrated [11,12,13,14]. Several studies have shown that depleting inflammatory immune cells leads to the rapid improvement in insulin sensitivity and glucose tolerance, which is associated with marked decreases in local and systemic inflammation in obese mice [15,16,17]. For example, depleting CD8^+^ T cells reduces macrophage infiltration into the adipose tissues and pro-inflammatory macrophage activation [15,18]. Reduced inflammation due to the absence of B cells in obese mice also ameliorates insulin resistance and glucose tolerance by inhibiting the activation of T cells and macrophages [17]. Conversely, suppression of the development of M1 macrophages by regulatory T cells in the adipose tissues reverses obesity-linked insulin resistance [19,20]. Therefore, it is evident that the activation of immune cells, such as pro-inflammatory macrophages, is a key contributor to adipose tissue inflammation.

Macrophages are known to have plasticity—they can switch between M1 and M2 states, which are associated with various diseases [21,22,23,24]. It has been suggested that during weight gain, macrophages undergo a phenotypic switch from an anti-inflammatory M2 phenotype to a pro-inflammatory M1 state, a conversion that is linked to the emergence of systemic insulin resistance [25]. However, several studies show that adipose tissue macrophages (ATMs) develop a mixed M1/M2 phenotype in obese mice and humans [26,27,28], suggesting that the ATM adopts more complex states in vivo. Whereas several pro-inflammatory cytokines, particularly IL-6, have been strongly associated with the development of M1 phenotypes of ATMs [25], myeloid cell IL-6 signaling can elicit the alternative activation of anti-inflammatory macrophages that limits the development of insulin resistance during obesity [29].

Myeloid cells respond to IL-6 through Jak/Stat3, which has been implicated in inflammatory disease and inflammation-promoted obesity-associated cancer [30,31]. Genetic ablation or pharmacologic inhibition of Stat3 in myeloid cells leads to the M1 phenotype and increased antitumor immune responses in several mouse tumor models [32,33,34,35]. It has also been shown that STAT3 activation promotes the M2 phenotype of human glioma-associated macrophages [36]. Previously, we have shown that Stat3 in T cells is essential in regulating the ratio of Th1 and regulatory T cells in the adipose tissue [20]. Ablating Stat3 in T cells reduces inflammation/obesity-induced insulin resistance, which is in part contributed to by the recruitment of IL-6-producing ATMs in obese mice [20]. These results suggest M1/M2 regulation by Stat3 is highly contextual. Nevertheless, the role of Stat3 in regulating macrophages/myeloid cells pertaining to obesity-induced insulin resistance remains unknown. Stat3 activation can be induced by many cytokines in addition to IL-6, growth factors, lipid metabolites, as well as toll-like receptors (TLRs) [37,38]. Furthermore, IL-6-induced Stat3 activation involves multiple members of Jak [37].

In this study, we investigated the role of Jak2 and Stat3 in myeloid cells/macrophages in modulating obesity-induced inflammation and insulin resistance. Our findings indicate an opposing role of Stat3 in regulating M1/M2 in obesity-associated diabetes vs. in the tumor microenvironment. We also demonstrated that Jak/Stat3 in myeloid cells can be targeted for inhibiting obesity/inflammation-induced insulin resistance.

## 2. Materials and Methods

### 2.1. Mice and Treatment

Mice care and experimental procedures were performed under pathogen-free conditions in accordance with established institutional guidance and approved protocols from the Institutional Animal Care and Use Committee at City of Hope. A 7–8-week-old male C57BL/5J (CD45.2, H-2kb) and a C57BL/6 congenic strain mice which express CD45.1 were obtained from Jackson Laboratories. Stat3^flox/flox^ mice were kindly provided by Dr. S. Akira (Osaka University) and Jak2^flox/flox^ mice by Dr. K-U Wagner (University of Nebraska Medical Center) [39]. Mx1-Cre mice with a C57BL/6J background (CD45.2, H-2kb) from Jackson Laboratories) were crossed with Stat3^flox/flox^ or Jak2^flox/flox^ mice to generate mice with Stat3 or Jak2 ablation in the myeloid compartment. Mice with Stat3^−/−^ or Jak2^−/−^ hematopoietic cells were generated by treating Mx1-Cre/Stat3^flox/flox^ or Mx1-Cre/Jak2^flox/flox^ mice with poly (I:C), as described previously [33]. B6.129P2-Lyz2tm1(cre)Ifo/J mice were obtained from The Jackson Laboratory. B6.129P2-Ly2ztm1(cre)Ifo/J mice were crossed with Stat3^flox/flox^ to generate mice with Stat3 ablation in myeloid cells.

To generate mouse models of a high fat diet (HFD)-induced obesity, male mice were fed a normal diet (ND) or HFD (60 kcal% fat; Research Diets) for up to 16 weeks, starting from 6−8 weeks of age. Mice were fasted for 16 h for the glucose tolerance test (GTT) before receiving 1 g/kg of body weight of glucose intraperitoneally. Circulating glucose levels were measured in blood collected from the tail vein at 30, 60, 90, and 120 min after the glucose injection using a glucometer (One Touch Ultra; Life Scan, Malvern, PA, USA). Insulin tolerance test (ITT) was performed in mice fasted for 4–6 h before an intraperitoneal administration of 0.75 U/kg of body weight of insulin (Humulin; Eli Lilly, Indianapolis, IN, USA). Blood insulin levels were determined at 30, 60, 90, and 120 min after the insulin injection.

To generate the CD45 chimeric mice, the C57BL/6 congenic mice which express CD45.1 were used as recipients and were given a lethal dose of 10 Gy radiation, followed by a tail vein injection of 10^7^ CD45.2 hematopoietic cells with or without Jak2 from Mx1-Cre/Jak2^flox/flox^ mice.

CpG conjugated siRNA was synthesized as previously described [34]. C57BL/6 mice were fed an HFD for 8 weeks, followed by a CpG-Luc siRNA or CpG-Stat3 siRNA treatment every other day for 4 weeks (the mice were maintained on an HFD during the CpG-Luc siRNA or CpG-Stat3 siRNA treatment).

### 2.2. Immune Cell Isolation and Flow Cytometry

Epididymal adipose tissue was collected and minced into small pieces (1–5 mm^3^) in PBS and centrifuged at 1000 g for 5 min to remove circulating cells. The floating adipose tissue was collected and digested with collagenase D (2 mg/mL) and DNase I (1 mg/mL, Roche, Indianapolis, IN, USA) for 30–45 min at 37°C. Enzymatically digested tissues were filtered through 70 μm cell strainers (Corning, Santa Barbara, CA, USA) and centrifuged at 600 g for 5 min. The pellet containing immune cells was collected, and cells were resuspended in PBS containing 2% FBS for flow cytometry. Cells were incubated with an anti-CD16/32 antibody (BioLegend, San Diego, CA, USA, #101301) to block Fc receptor, followed by staining with fluorophore-conjugated antibodies (CD45, CD11b, F4/80, CD11c, and CD206 from Biolegend and eBioscience) for 15 min on ice. Data was collected on BD LSRFortessa and analyzed by FlowJo software (FlowJo 10.8.1.), as described previously [40]. Gating strategies for macrophages are shown in Figure S1.

### 2.3. Histology and Immunohistochemistry (IHC)

The visceral adipose tissue (VAT) fat pads, liver, and pancreas were fixed in 10% buffered formalin for 24 h, dehydrated, and embedded in paraffin. Sections were cut in 5 μm thickness and stained with H&E solution. For IHC, paraffin-embedded VAT sections were de-paraffinized and incubated with a rat anti-mouse F4/80 antibody (Serote, 1:100) for 1 h at RT. Nuclei were counterstained with hematoxylin. Sections were visualized with an Olympus AX70 (Olympus, Bartlett, TN, USA) automated upright microscope. Crown-like structures (CLSs) and adipocyte cell diameter were determined as described [20]. For insulin staining, formalin-fixed, paraffin-embedded pancreatic tissues were cut into 5 μm thickness with a distance of 75 μm between each section. Five sections were stained with the Guinea Peg anti-insulin antibody (DAKO, Carpinteria, CA, USA #A0564, 1:5000) for 1 h at RT and hematoxylin and visualized with an Olympus AX70 automated upright microscope. Islet areas were measured from antibody-stained sections using Image-Pro Premier 9.0 software (Media Cybernetics, Rockville, MD, USA).

### 2.4. Free Fatty Acid (FFA) Treatment

FFA solution was prepared as described previously [41,42]. Briefly, 100 mM palmitate stock solution was prepared in 0.1 M NaOH at 70 °C in a shaking water bath. A total of 5.26% FFA-free BSA solution was prepared in H_2_O. To prepare 5 mM palmitate/5% BSA stock solution, 50 μl of the 100 mM FFA solution was added dropwise to 950 μl 5.26% BSA solution at 55 °C in a shaking water bath. The 5 mM palmitate/5% BSA solution was cooled down to room temperature and stored at −80 °C. Peritoneal macrophages were isolated from mice and incubated with 0.5 mM palmitate/0.5% BSA for 16 h or 48 h at 37 °C.

### 2.5. Measurement of FFA, Insulin, and Triglyceride Concentration

Circulating FFA levels were measured by a Free Fatty Acid Quantification Kit (Abcam, Cambridge, UK #ab65341). Plasma insulin levels were determined by ELISA according to the manufacturer’s instruction (Crystal Chem, Elk Grove Village, IL 60007, USA, #90080). Circulating triglycerides were measured by a commercial assay kit available from Cayman Chemical (Ann Arbor, MI, USA, #10010303).

### 2.6. Measurement of IL-6 and CCL2 Concentration

The concentrations of IL-6 and CCL2 in the cultured supernatant were measured by ELISA assay. ELISA kits were purchased from Biolegend (#431304 and # 432704).

### 2.7. Quantitative Real-Time PCR

Total RNAs from VAT and immune cells were purified using the RNeasy kit following the manufacturer’s instruction (Qiagen, Germantown MD, USA, #74534). RNA (0.5 to 1 μg) was reverse-transcribed to cDNA using the iScript cDNA Synthesis Kit (Bio-Rad, Hercules, CA, USA, #1708840), and real-time RT-PCR reactions were performed using Bio-Rad SYBR Green Supermix, as described previously [43]. Specific primers for mouse *Gapdh*, *Il6*, *Ccl2*, and *Emr1* (F4/80) were purchased from SA Bioscience (Frederick, MD, USA). For *Jak2, Cd163,* and *Retnla*, Q-PCR reactions were performed using the following primers: mouse *Jak2* 5′AAC TGT TTT CCC TCC CCA GAAG3′ (Forward), 5′TAAGGCAGGCCATTCCCATCTA3′ (Reverse); mouse *Cd163* 5′ TGC AGG TGT TAT CTG CTG CGA GTT 3′ (Forward), 5′ AGG TAT GGG TTT CAC AGT CCC GTT3′ (Reverse); and mouse *Retnla* 5′ CTT GTG GCT TTG CCT GTG GAT CTT 3′ (Forward), 5′ TGG TCC AGT CAA CGA GTA AGC ACA3′(Reverse).

### 2.8. Phospho-ELISA

Mouse VAT-associated CD11b^+^ myeloid cells were FACS sorted by the Aria III Sorter (BD, Qume Drive, San Jose, CA, USA). Cell pellets were collected and lysed to determine p-Stat3 or p-Jak2 levels by ELISA (Invitrogen, Carlsbad, CA, USA, # KHO0481 and # KHO5621).

### 2.9. Statistical Analysis

Statistical analyses were performed using GraphPad Prism 10 software (GraphPad Software, Boston, MA, USA). Student’s *t*-test was used to determine the significant difference between the means of two groups. ANOVA was used to assess the statistical significance of the means of three or more groups. Statistical significance values were set as * *p* < 0.05, ** *p* < 0.01, and *** *p* < 0.001. Data are shown as a mean ± SEM. *p* value, and *n* can be found in the main and supplemental figure legends.

## 3. Results

### 3.1. Myeloid Cell Jak2 Promotes Obesity-Induced Insulin Resistance

To test the role of myeloid cell intrinsic Jak2–Stat3 signaling inducing obesity-associated insulin resistance, we generated control (Mx1-Cre/Jak2^+/+^) mice and mice with the hematopoietic cell ablation of Jak2 (Mx1-Cre/Jak2^−/−^). Genotype analysis demonstrated the disruption of the Jak2 in CD11b^+^ myeloid cells isolated from the Jak2-deficient mice (Jak2^−/−^) but not the control mice (Jak2^+/+^) (Figure S2A). This was confirmed through an immunoblotting analysis of Jak2 protein expression in myeloid cells isolated from the spleen and VAT of mice with the Jak2^+/+^ and Jak2^−/−^ myeloid compartment (Figure S2B).

**Figure 1:**
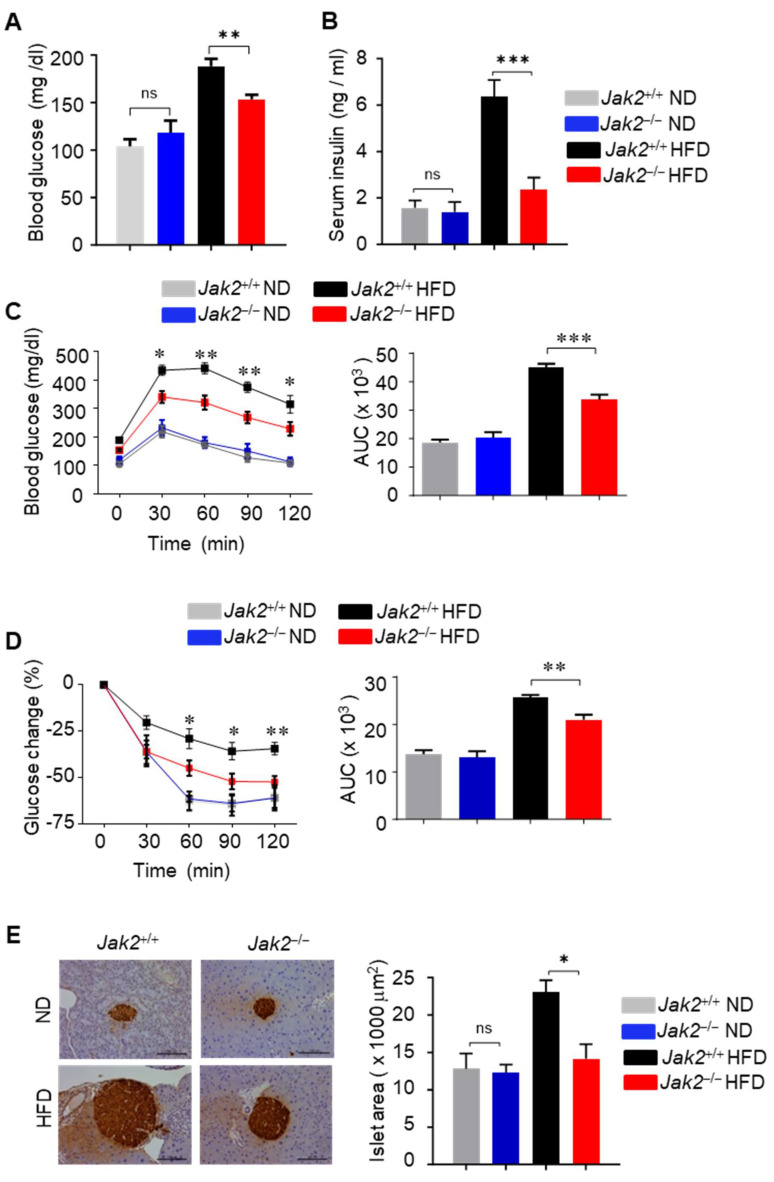
Ablating *Jak2* in myeloid compartment improves insulin sensitivity in DIO mice. (**A**) Blood glucose levels from mice on ND or HFD for 16 weeks. Mice were fasted overnight before glucose test; mean ± SEM, *n* = 8; and ** *p* < 0.01. ns = non-significant. (**B**) ELISA quantifying the level of circulating insulin in serum of ND- or HFD-fed mice with *Jak2*^+/+^ and *Jak2*^−/−^(*Mx1-Cre*/*Jak2*^−/−^) myeloid compartment; mean ± SEM, *n* = 13; and *** *p* < 0.001. ns = non-significant. (**C**) Glucose tolerance tests (GTTs) performed on mice with *Jak2*^+/+^ and *Jak2*^−/−^ myeloid compartment on ND or HFD for 16 weeks; mean ± SEM, *n* = 8; * *p* < 0.05; and ** *p* < 0.01. The area under curve (AUC) was calculated based on the GTT data, *** *p* < 0.001. (**D**) Insulin tolerance tests (ITT) performed on mice with *Jak2*^+/+^ and *Jak2*^−/−^ myeloid compartment fed on ND or HFD for 16 weeks. Glucose levels in ITTs were expressed as % change relative to time 0 before insulin injection; mean ± SEM, *n* = 8; * *p* < 0.05; and ** *p* < 0.01. AUC is shown on the right of this panel, ** *p* < 0.01. (**E**) Microscopy analysis shows insulin staining of islet areas in the pancreas of HFD-fed mice with *Jak2*^+/+^ compartment compared to their *Jak2*^−/−^ counterpart. Sections were stained with antibody to insulin; scale bars, 100 μm (left). A graph depicts insulin-positive area size per section; mean ± SEM, *n* = 6 mice; and * *p* < 0.05 (right). ns = non-significant.

Diet-induced obesity has been strongly associated with hyperinsulinemia and insulin resistance [1]. ND-fed mice with the Jak2^+/+^ and Jak2^−/−^ myeloid compartment displayed similar levels of glucose and insulin in the blood (Figure 1A,B). Importantly, Jak2 deficiency in myeloid cells prevented HFD-induced hyperinsulinemia and hyperglycemia and improved both insulin and glucose tolerance as compared with that of HFD-fed Jak2^+/+^ mice (Figure 1A–D). Pancreatic islets in ND-fed Jak2^+/+^ and Jak2^−/−^ mice were morphologically similar (Figure 1E). HFD-induced β cell proliferation and islet hypertrophy were only observed in Jak2^+/+^ mice but not in Jak2^−/−^ mice (Figure 1E). These data are consistent with the results that HFD-fed Jak2^−/−^ mice exhibit improved insulin sensitivity as compared with that of HFD-fed Jak2^+/+^ mice.

**Figure 2:**
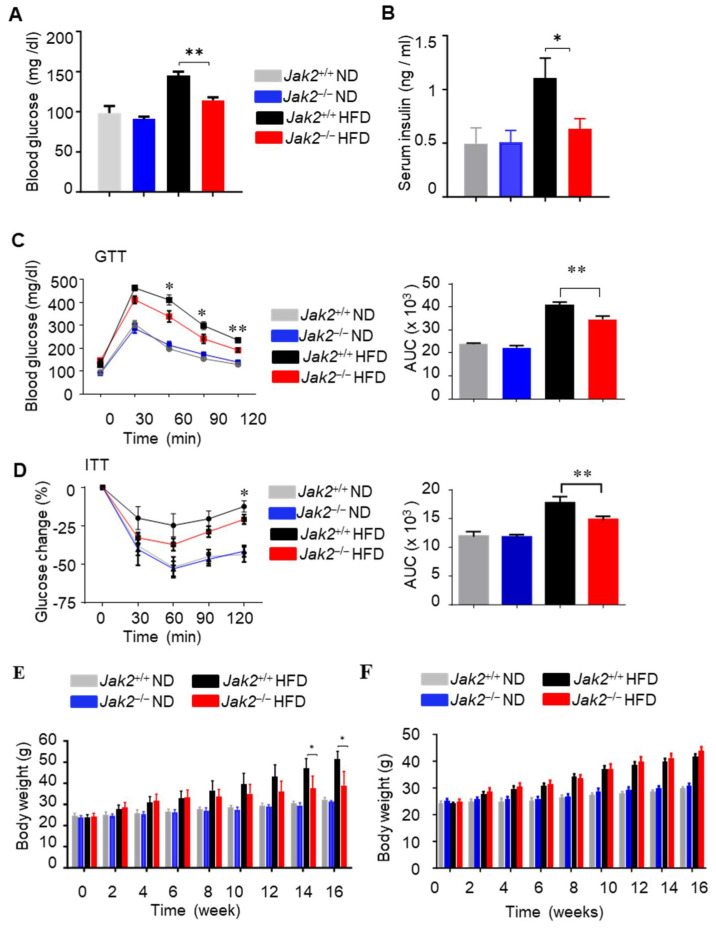
Ablating *Jak2* in myeloid compartment improves insulin sensitivity in chimeric DIO mice. (**A**) Blood glucose levels from *Jak2*^+/+^ and *Jak2*^−/−^ (*Mx1-Cre*/*Jak2*^−/−^) chimeric mice (mice receiving *Jak2*^+/+^ and *Jak2*^−/−^ hematopoietic cell transplantation) on ND or HFD for 16 weeks. Mice were fasted overnight before blood sample collection; mean ± SEM, *n* = 8; and ** *p* < 0.01. (**B**) ELISA quantifying the level of circulating insulin in serum of ND- or HFD-fed *Jak2*^+/+^ and *Jak2*^−/−^ chimeric mice; mean ± SEM, *n* = 10; and * *p* < 0.05. (**C**) Glucose tolerance tests (GTTs) performed on *Jak2*^+/+^ and *Jak2*^−/−^ chimeric mice on ND or HFD for 16 weeks; mean ± SEM, *n* = 8; * *p* < 0.05; and ** *p* < 0.01. The area under curve (AUC) was calculated based on the GTT data, ** *p* < 0.01. (**D**) Insulin tolerance tests (ITTs) performed on *Jak2*^+/+^ and *Jak2*^−/−^ chimeric mice on ND or HFD for 16 weeks. Glucose levels in ITTs were expressed as % change relative to time 0 before insulin injection; mean ± SEM, *n* = 8; * *p* < 0.05; and ** *p* < 0.01. AUC is shown on the right of this panel, ** *p* < 0.01. (**E**) The body mass of *Jak2*^+/+^ and *Jak2*^−/−^(*Mx1-Cre*/*Jak2*^−/−^) mice fed an ND and HFD was measured at the indicated times; mean ± SEM, *n* =4–7; and * *p* < 0.05. (**F**) The body mass of Jak2^+/+^ and Jak2^−/−^ chimeric mice fed ND and HFD was measured at the indicated times (mean ± SEM; *n* = 8). No statistically significant differences between chimeric Jak2^+/+^ and Jak2^−/−^ mice were detected (*p* > 0.05).

To exclude the effects of Jak2 ablation in hepatic cells, which occurs in Mx-1 Cre mice, we performed an adoptive transfer of hematopoietic cells. Specifically, wild-type C57BL/6 congenic mice (CD45.1) were lethally irradiated and reconstituted with hematopoietic cells with or without Jak2 alleles in myeloid cells from Jak2^flox/flox^ and Mx1-Cre/Jak2^flox/flox^ mice (CD45.2) (Figure S3A). Flow cytometric analysis showed that, 6 weeks after bone marrow transplantation, bone marrow was efficiently reconstituted with white blood cells, which displayed the donor genotype (CD45.1) (Figure S3B). Western blotting showed Jak2 protein expression in CD11b^+^ myeloid cells from chimeric Jak2^+/+^ mice, but not those from chimeric Jak2^−/−^ mice (Figure S3C top), and both chimeric Jak2^+/+^ and chimeric Jak2^−/−^ mice exhibited Jak2 protein expression in hepatocytes (Figure S3C bottom). The obese chimeric mice with Jak2^−/−^ hematopoietic cells displayed lower plasma insulin and glucose levels compared to their counterparts with wild-type Jak2 (Figure 2A,B). Moreover, the mice receiving Jak2^−/−^ hematopoietic cells and maintained for 16 weeks on an HFD had improved glucose and insulin tolerance compared to their counterpart group transferred with wild-type hematopoietic cells (Figure 2C,D). To assess the effect of body weight on insulin resistance and glucose tolerance, we compare the body weight of Mx1-Cre/Jak2^+/+^ and Mx1-Cre/Jak2^−/−^ mice. These mice exhibited similar increased weights after being fed an HFD for 12 weeks, although there was some reduction in obesity-associated mass detected in the Jak2^−/−^ mice compared with the Jak2^+/+^ mice after 14 weeks of an HFD (Figure 2E). Meanwhile, no evident differences in food intake were observed between the two groups of mice (Figure S2C). Importantly, HFD-fed Jak2^+/+^ and Jak2^−/−^ chimeric mice had similar body weights (Figure 2F), while both groups on an HFD exhibited increased body weight gain relative to those on an ND (Figure 2F). These results further indicate that the myeloid cell Jak2 promotes obesity-associated insulin resistance independent of body mass per se.

#### 3.1.1. Jak2 Promotes M1 Polarization of ATMs During Obesity

**Figure 3:**
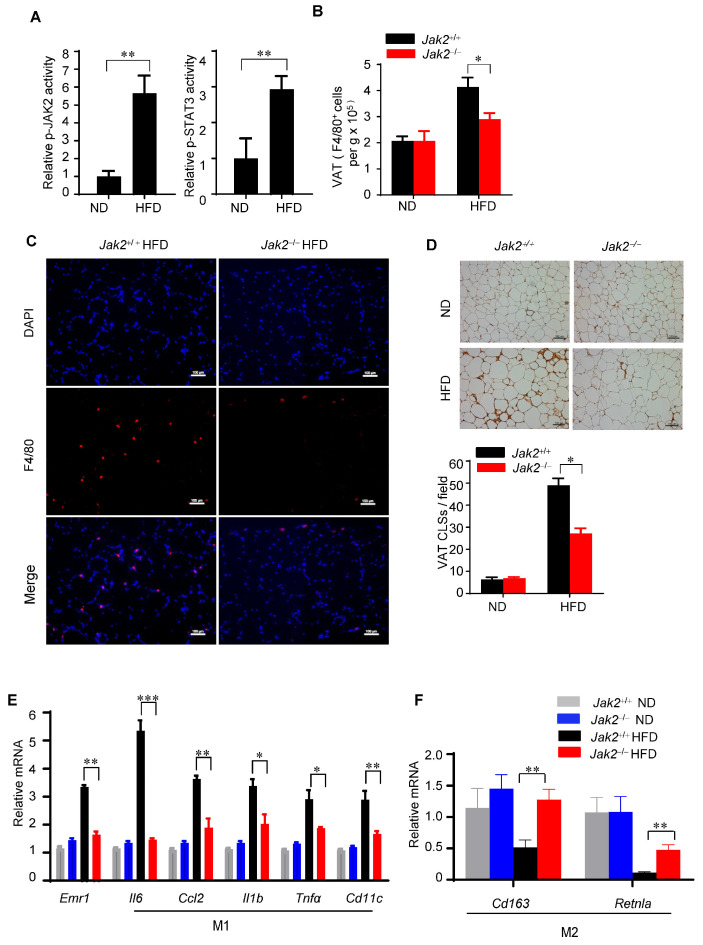
Jak2 promotes M1 polarization of adipose tissue macrophages. (**A**) ELISA quantifying p-Stat3 and p-JAK2 in CD11b^+^ myeloid cells isolated from the stromal vascular fraction (SVF) of epididymal adipose tissue of ND- or HFD-fed mice. Relative p-Stat3 and p-JAK2 activities were normalized to total protein amount; mean ± SEM, *n* = 3 mice. ** *p* < 0.01. (**B**) Flow cytometry to detect the total number of F4/80^+^ macrophages infiltrating visceral adipose tissue (VAT) of mice with *Jak2*^+/+^ and *Jak2*^−/−^ myeloid compartment on ND or HFD; mean ± S EM, *n* = 6 mice each group. * *p* < 0.05. (**C**) Immunofluorescent staining showing the degree of F4/80^+^ macrophage infiltration in the adipose tissue of Jak2^+/+^ and Jak2^−/−^ mice on an ND or HFD. Scale bars, 100 μm. (**D**) Microscopic images of VAT sections showing crown-like structures (CLSs) of F4/80^+^ macrophages in VAT of mice with *Jak2*^+/+^ and *Jak2*^−/−^ compartment fed an ND or HFD (top); scale bars, 100 μm. Analysis and quantification of CLSs in VAT sections; mean ± SEM, *n* = 4 mice each group. * *p* < 0.05 (bottom). (**E**,**F**) The relative expressions of mRNA associated with M1-polarized macrophages (**E**) and M2-polarized macrophages (**F**) were measured by quantitative RT-PCR assays (mean ± SEM, *n* = 4, and each sample was pooled from 3 mice). Total RNA was isolated from VAT from ND- or HFD-fed *Jak2*^+/+^ and *Jak2*^−/−^ mice. * *p* < 0.05; ** *p* < 0.01; and *** *p* < 0.001.

We found that both Jak2 and Stat3 were highly activated in the adipose tissue myeloid cells of HFD-fed wild-type mice relative to their ND-fed wild-type mice counterparts (Figure 3A). No differences in the number of adipose tissue immune cells were detected in *Jak2*^+/+^ and *Jak2*^−/−^ mice fed with the ND. In contrast, feeding them an HFD led to an increase in the number of stromal vascular fraction (SVF) cells in *Jak2*^+/+^ mice relative to *Jak2*^−/−^ mice (Figure S4A). The number of adipose tissue macrophages was significantly reduced in *Jak2*^−/−^ mice relative to *Jak2*^+/+^ mice receiving an HFD (Figure 3B,C). These data indicate that Jak2 in myeloid cells promotes the HFD-induced accumulation of adipose tissue macrophages.

The majority of infiltrated macrophages in adipose tissues are found around dead adipocytes, thereby forming CLSs [44]. We performed immunostaining to examine adipose tissue macrophages in Jak2^+/+^ and Jak2^−/−^ mice. Ablating Jak2 in the myeloid compartment decreased CLSs in obese mice (Figure 3D). Because M1-polarized macrophages are implicated in the development of insulin resistance, we examined an adipose tissue gene expression profile to test whether Jak2 influences the polarization of adipose tissue macrophages. This analysis demonstrated that myeloid cell Jak2 deficiency decreased the expression of genes associated with M1 polarization and increased those associated with M2 polarization in the adipose tissue macrophages of HFD-fed mice (Figure 3E,F). These data confirm that Jak2 expression in myeloid cells promotes both adipose tissue macrophage accumulation and M1 polarization in HFD-fed mice.

#### 3.1.2. Jak2–Stat3 Pathway Mediates FFA-Induced Inflammation

**Figure 4:**
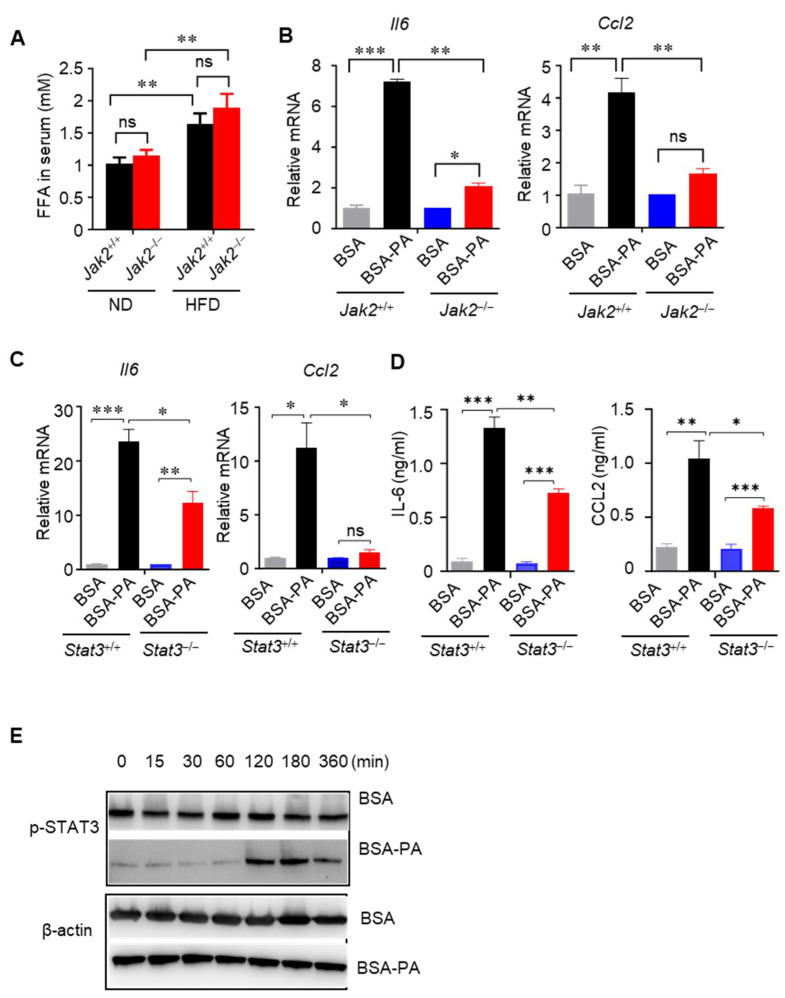
Jak2/Stat3 signaling contributes to FFA-mediated induction of pro-inflammatory cytokines in macrophages. (**A**) HFD-fed *Jak2*^+/+^ and *Jak2*^−/−^ (*Mx1-Cre*/*Jak2*^−/−^) mice were fasted overnight before measuring fasting serum FFA levels (mean ± SEM, *n* = 14 mice). ** *p* < 0.01. ns = non-significant. (**B,C**) Peritoneal macrophages from mice with *Jak2*^+/+^ and *Jak2*^−/−^ (**B**) or *Stat3*^+/+^ and *Stat3*^−/−^ myeloid compartment (**C**) were incubated with 0.5% BSA or 0.5% BSA-conjugated 0.5 mM palmitate (PA) for 18 h before RNAs were isolated. The relative expression of the indicated pro-inflammatory M1 marker genes was measured by quantitative RT-PCR assays; mean ± SEM, *n* = 3, * *p* < 0.05, ** *p* < 0.01, and *** *p* < 0.001. Data are from three experiments. ns = non-significant. (**D**) Peritoneal macrophages from mice with *Stat3*^+/+^ and *Stat3*^−/−^ myeloid compartment were incubated with 0.5% BSA or 0.5% BSA-conjugated 0.5 mM palmitate for 48 hrs. The concentration of IL-6 and CCL2 in the supernatants were detected by ELISA. Mean ± SEM, *n* = 3 experiments, * *p* < 0.05, ** *p* < 0.01, and *** *p* < 0.001.(**E**) Immunoblot analysis to detect p-Stat3 levels in peritoneal macrophages incubated with 0.5% BSA or 0.5% BSA-conjugated 0.5 mM palmitate for indicated time points. Cells from 3 to 4 C57BL/6 wild-type mice were pooled together to prepare cell lysates for analysis.

Obesity-induced FFA elevation contributes to the development of insulin resistance by inflammation [45]. Although the blood concentrations of FFAs in HFD-fed *Jak2*^+/+^ and *Jak2*^−/−^ mice were similar, HFD-fed *Jak2*^+/+^ and *Jak2*^−/−^ mice contained increased levels of FFAs in circulation compared to those of the ND-fed mice (Figure 4A). In addition, feeding them an HFD led to an increase in the number of macrophages and the expression of genes associated with M1 polarization in *Jak2*^+/+^ mice relative to *Jak2*^−/−^ mice (Figure 3). To test whether FFA-associated inflammation regulated by Jak2 impacts adipose tissue inflammation driven by macrophages, we isolated macrophages from *Jak2*^+/+^ and *Jak2*^−/−^ mice. Treatment of wild-type macrophages with FFAs increased the expression of pro-inflammatory M1 cytokines such as *Il6* and *Ccl2* (Figure 4B). *Jak2* ablation reduced the expression of these cytokine genes in FFA-stimulated macrophages (Figure 4B). Furthermore, *Stat3^-/-^* macrophages from *Mx1-Cre/Stat3^flox/flox^* mice also decreased the expression of *Il6* and *Ccl2* after FFA stimulation, as compared with those from wild-type mice (Figure 4C,D). The expression of IL-6 and MCP-1 depends on Stat3 activity in immune cells [20]; we therefore tested that the increased cytokine expression in macrophages in response to FFAs is a consequence of Stat3 activation. Indeed, we observed that Stat3 phosphorylation was highly elevated in macrophages from the C57BL/6 wild-type mice upon FFAs treatment (Figure 4E). Collectively, these data indicate that obesity/FFAs contribute to M1 cytokine expression in macrophages through activating Jak2/Stat3 signaling.

#### 3.1.3. Stat3 Is a Target to Ameliorate Obesity-Induced Insulin Resistance

**Figure 5:**
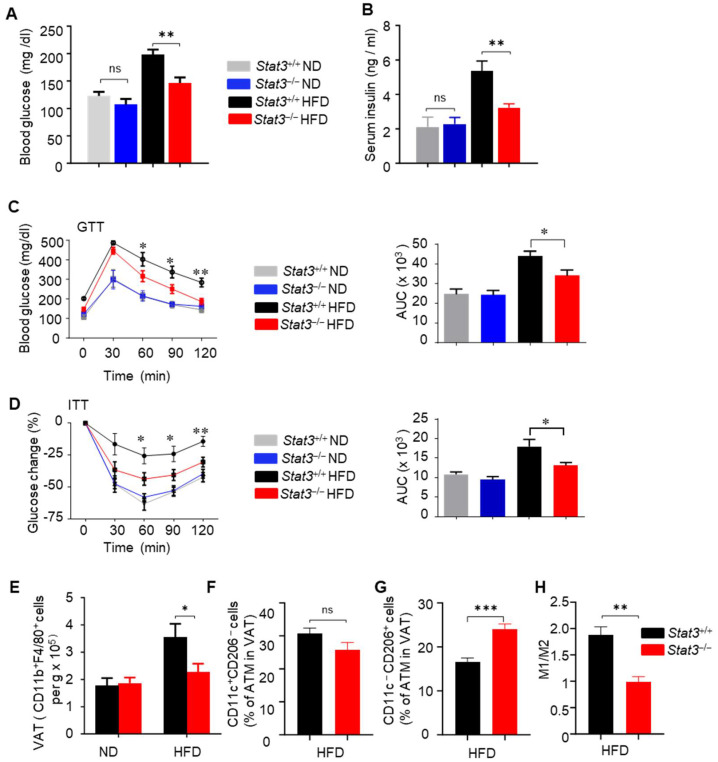
Ablating *Stat3* in macrophages improves insulin sensitivity in DIO mice. (**A**) Blood glucose levels from mice with *Stat3*^+/+^ and *Stat3*^−/−^ myeloid cells (*Lyz-Cre*) on an ND or HFD for 16 weeks. Mice were fasted overnight before glucose test; mean ± SEM, *n* = 8; and ** *p* < 0.01. ns = non-significant. (**B**) ELISA quantifying the level of circulating insulin in serum of ND- or HFD-fed mice with *Stat3*^+/+^ and *Stat3*^−/−^ myeloid cells; mean ± SEM, *n* = 10; and ** *p* < 0.01. ns = non-significant. (**C**) Glucose tolerance tests (GTTs) performed on mice with *Stat3*^+/+^ and *Stat3*^−/−^ myeloid compartment on an ND or HFD for 16 weeks; mean ± SEM, *n* = 8; * *p* < 0.05; and ** *p* < 0.01. The area under curve (AUC) was calculated based on the GTT data, * *p* < 0.05. (**D**) Insulin tolerance tests (ITTs) performed on mice with *Stat3*^+/+^ and *Stat3*^−/−^ myeloid compartment on an ND or HFD for 16 weeks. Glucose levels in ITTs were expressed as % change relative to time 0 before insulin injection; mean ± SEM, *n* = 8; * *p* < 0.05; and ** *p* < 0.01. AUC is shown on the right of this panel, ** *p* < 0.05. (**E–H**) Flow cytometric analysis to quantitate CD11b^+^F4/80^+^ macrophages, CD11c^+^CD206*^−^* (M1) and CD11c*^−^*CD206^+^ (M2) as well as the ratio of M1/M2 in the stromal vascular fraction (SVF) of epididymal adipose tissues derived from indicated mice.; mean ± SEM, *n* = 6; and * *p* < 0.05, ** *p* < 0.01, and *** *p* < 0.001. ns = non-significant. Data are representative of two experiments.

Given the importance of Stat3 in mediating Jak2-driven insulin resistance and inflammation in obese mice (Figure 4C,D), we generated control (*Lyz-Cre*/*Stat3*^+/+^) mice and mice with macrophage cells lacking functional *Stat3* alleles (*Lyz-Cre*/Stat3^−/−^). We then examined whether Stat3 in myeloid cells mediates obesity-induced insulin resistance. ND-fed mice with functional *Stat3*^+/+^ and *Stat3*^−/−^ in macrophages displayed similar insulin and glucose tolerance and blood concentrations of glucose and insulin (Figure 5A–D). Feeding an HFD to mice with *Stat3*^+/+^ myeloid cells led to hyperglycemia, hyperinsulinemia, and an intolerance to both glucose and insulin (Figure 5A–D). In contrast, mice with *Stat3*^−/−^ myeloid cells did not develop HFD-induced hyperglycemia and hyperinsulinemia, insulin resistance, and glucose tolerance. The protection of mice with a macrophage-specific *Stat3* deficiency against insulin resistance is associated with the reduced tissue infiltration by macrophages (Figure 5E). In addition to the number of ATMs infiltration, M1/M2 polarity is associated with insulin resistance. M1-polarized macrophages with a higher M1/M2 ratio are implicated in the development of insulin resistance [46]. Therefore, we assessed the M1 and M2 populations of ATMs in mice with Stat*3*^+/+^ and *Stat3*^−/−^ macrophages. Stat3 ablation in macrophages reduced ATMs expressing M1 markers (CD11b^+^F4/80^+^CD11c^+^CD206*^−^*), with a significantly lower M1/M2 ratio in the adipose tissues of the HFD-fed mice (Figure 5F–H). Meanwhile, the mice with *Stat3*^+/+^ and *Stat3*^−/−^ macrophages exhibited similar obesity when fed an HFD (data not shown).

**Figure 6:**
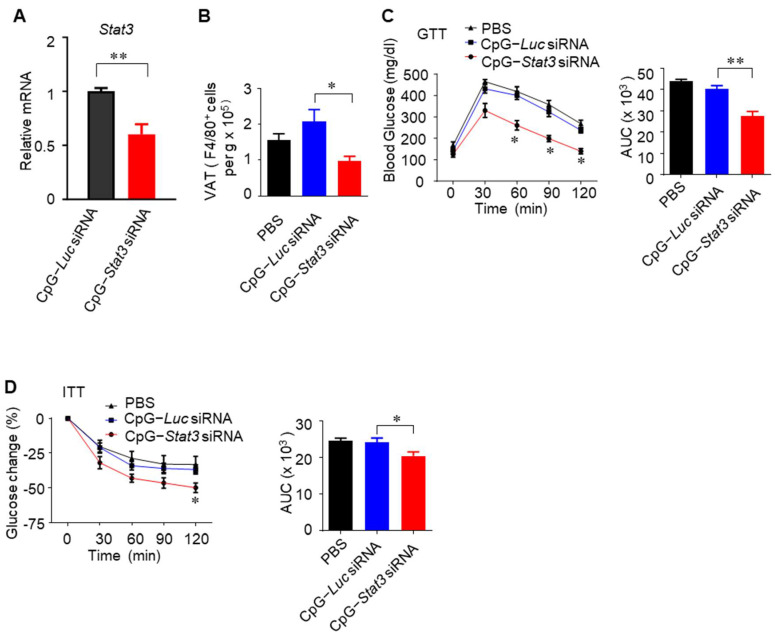
Targeting *Stat3* in myeloid compartment reverses obesity-induced insulin resistance. (**A**) Real time RT-PCR showing *Stat3* transcription level in adipose tissue CD11b^+^ myeloid cells from HFD-fed mice, treated with the indicated CpG-conjugated siRNAs; ** *p* <0.01. (**B**) Flow cytometry to detect the number of F4/80^+^ macrophages in the adipose tissue of HFD-fed mice treated as indicated; * *p* < 0.05. (**C**,**D**) GTT (**C**) and ITT (**D**) performed on HFD-fed mice treated as indicated; * *p* < 0.05, ** *p* < 0.01. AUC is shown on the right. In all cases, means ± SEM, *n* = 6.

We further tested whether targeting *Stat3* in myeloid cells could inhibit macrophage infiltration into the VAT and reverse obesity-induced insulin resistance. Recently, we have developed a novel siRNA delivery approach based on CpG targeting TLR9-expressing cells such as myeloid cells, leading to potent antitumor immune responses [34]. Treatment of HFD-fed C57BL/6 mice with CpG-*Stat3* siRNA efficiently reduced the *Stat3* transcription level in myeloid cells infiltrating adipose tissues (Figure 6A). As a result, the number of macrophages in the adipose tissue of obese mice was greatly decreased (Figure 6B). Obese mice receiving CpG-*Stat3* siRNA treatment exhibited an improved glucose tolerance as well as insulin sensitivity (Figure 6C,D). These findings reinforce our hypothesis that myeloid cell Jak2/Stat3 signaling is crucial for the development of obesity-induced insulin resistance and inflammation and can be effective therapeutic targets for type 2 diabetes.

## 4. Discussion

ATM inflammation plays a key role in obesity-associated insulin resistance and type 2 diabetes [5,10,47]. Using mice with a *Jak2* deficiency in the myeloid compartment and *Stat3* ablation in myeloid cells, we identify cell-intrinsic Jak2/Stat3 signaling as a critical driver of the pro-inflammatory ATM phenotype in obesity. Our demonstration that Jak2/Stat3 is persistently activated in the fat-laden adipose tissue microenvironment has important implications for understanding how pro-inflammatory ATM phenotypes are amplified during obesity. Several pro-inflammatory cytokines such as TNFα and IL-6 induce adipocyte lipolysis, leading to an elevated level of circulating FFAs in obese mice [48,49]. We further show that FFAs induce Jak2/Stat3 activation in macrophages. Based on these observations, our study suggests that Jak2/Stat3 links cytokines and lipid metabolites to shaping the repertoire of inflammatory phenotypes in macrophages. Although how FFAs activate Jak2/Stat3 requires further investigation, the importance of lipid metabolite sphingosine-1-phosphate (S1P) and its cognate receptor S1PR1 in inducing persistent Jak2/Stat3 activation in myeloid cells has been demonstrated [50]. In addition to Stat3, Stat5 can be activated by Jak2. Although Jak–Stat5 signaling favors the pro-inflammatory polarization of macrophages [51], there is no evidence directly linking Stat5 activation in macrophages to inflammation related insulin resistance [52]. However, CXCL5 in insulin-sensitive tissues, such as muscles, mediates insulin resistance through the Jak2/Stat5 signaling pathway [53].

Metabolic dysfunction and excess FAs impose challenges for ATMs. Obesity leads to the continuous and excessive exposure of ATMs to FFAs, particularly saturated FFAs such as palmitate [54,55], which our data suggest plays a key role in triggering the metabolic activation of macrophages. Jak2/Stat3 promotes myeloid cell proliferation and survival [50], and thus it may enable ATMs to appropriately buffer their environment from excess lipids while maintaining cell viability. Our findings indicate that saturated FFAs increase the expression of pro-inflammatory cytokines through Jak2/Stat3 in macrophages. A previous study shows that IL-6 trans-signaling modulates TLR4-dependent inflammatory responses via Stat3 [56]. As FFAs can bind to inflammatory receptors such as TLR4 that drive cytokine production [57], it is possible that Jak2/Stat3 mediates FA binding to TLR4 to potentiate adipose tissue inflammation during obesity. On the other hand, the excessive accumulation of FAs within the macrophages is shown to promote the generation of anti-inflammatory macrophage phenotypes, attenuating inflammation [58].

Jak2/Stat3 in tumor-associated macrophages/myeloid cells is also crucial for inducing cancer-promoting inflammatory immune responses while inhibiting antitumor immune responses [33]. The presence of a mixed macrophage phenotype in obese mice has been shown [25,26]. Moreover, feeding mice for extended periods can reverse the obesity-induced shift in ATMs to an anti-inflammatory phenotype from a pro-inflammatory phenotype [27]. Therefore, the balance between pro- and anti-inflammatory processes may determine the overall response of ATMs to metabolic insults. However, whether the shifts in M1 and M2 balance affect insulin resistance during metabolic challenges such as excess lipids remains to be determined. Instead, as suggested by our findings for both the genetic ablation of macrophage Stat3 and silencing Stat3 through CpG-*Stat3*siRNA in the myeloid compartment, the signaling pathway within macrophage/myeloid cells rather than M1/M2 is more critical for obesity/inflammation-mediated insulin resistance. It has become increasingly evident that obesity/excess lipids are linked to increased cancer incidence and resistance to various cancer therapies. Although further investigations are required to directly address the mechanisms underlying obesity/inflammation-mediated pro-oncogenic effects, our findings that Jak2/Stat3 in myeloid/macrophages promotes the accumulation of FFAs, which in turn activate Jak2/Stat3, provide a potential molecular mechanism that links obesity with cancer.

In addition to FFAs, adipose tissues produce various hormones, growth factors, and cytokines that activate JAKs [59]. Our data provides evidence that ablating *Jak2 or Stat3* in myeloid cells reverses the phenotype of obesity/ATM inflammation-linked insulin resistance in vivo*. Jak2* or *Stat3* may therefore represent potential therapeutic targets for improving obesity-linked insulin resistance. However, both JAK2 and STAT3 play essential roles in the peripheral metabolic organs such as the liver and pancreas [60,61]. As a result, the systemic inhibition of JAK2/STAT3 may lead to undesirable metabolic consequences due to off-target effects in these organs. In contrast, targeting Stat3 in myeloid cells using our CpG-conjugated siRNA sufficiently reverses insulin resistance in vivo, supporting the idea that selective inhibition of *Jak2* or *Stat3* in myeloid cells may offer a safer and more specific therapeutic approach for treating obesity-related insulin resistance.

## 5. Conclusions

Our study on *Lyz-Cre* mice in which *Stat3* ablation specifically occurred in the macrophage showed the importance of Stat3 in macrophages in mediating obesity-induced inflammation and insulin resistance. Both macrophages and B cells are important in mediating obesity-associated insulin resistance [17,62]. We show through both *Mx-1 Cre* mice and CpG-*Stat3* siRNA that blocking *Stat3* in B and myeloid cells can effectively reduce insulin resistance. CpG-*Stat3* siRNA has been shown in multiple tumor models to be an effective immunomodulator for cancer therapy, and it is scheduled for phase 1 clinical trials in cancer patients. The current study raises the possibility of the future use of CpG-*Stat3* siRNA to ameliorate insulin resistance. Our previous report demonstrated Stat3 as a target in T cells in reducing obesity-mediated insulin resistance [20]. These findings, taken together, suggest that Stat3 in immune cells is an effective target for preventing and/or treating type 2 diabetes.

## Data Availability

The original contributions presented in the study are included in the article/Supplementary Materials. Further inquiries can be directed to the corresponding authors.

## Supplementary Materials

The following supporting information can be downloaded at: https://www.mdpi.com/article/10.3390/cells14151194/s1.
