# Supplementary material for: JAK2/STAT3 Signaling in Myeloid Cells Contributes to Obesity-Induced Inflammation and Insulin Resistance
Source: Cells. 2025 Aug 2;14(15):1194. doi: 10.3390/cells14151194 (PMC12346878; doi:10.3390/cells14151194)
Supplement: Supplementary file 1 [file cells-14-01194-s001.zip › cells-3761457-supplementary.pdf]

A

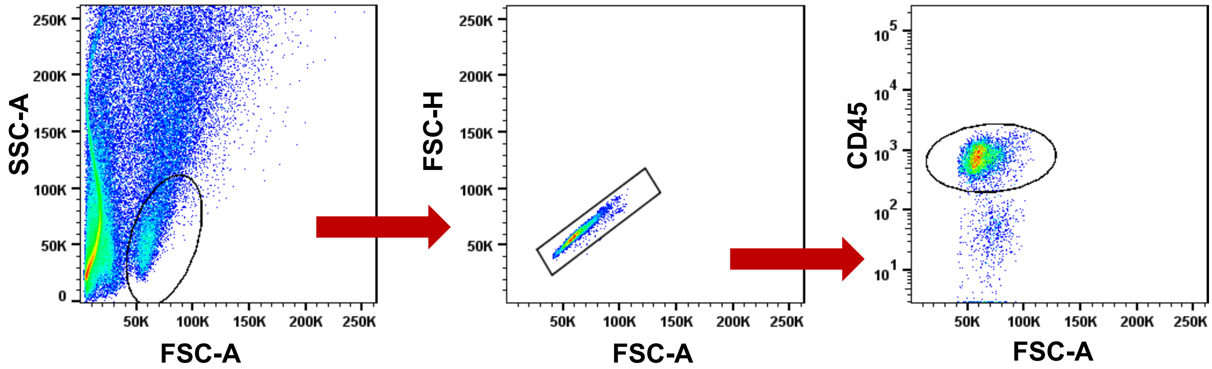

B

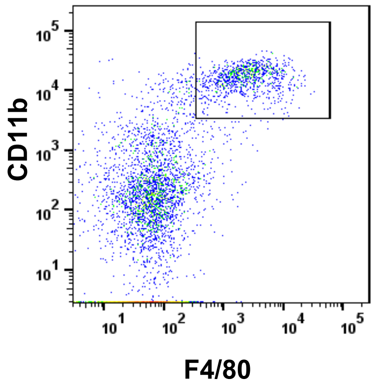

Supplemental Figure S1. Gating strategy of macrophages in the adipose tissue. (A) First, the cell debris (SSC-A/FSC-A), doublets (FCS-A/FSC-H) cells and $CD45^-$ none immune cells were excluded from all analyses, as shown for one representative FACS staining. (B) Gating of macrophages ($CD11b^+F4/80^+$) in $CD45^+$ immune cells.

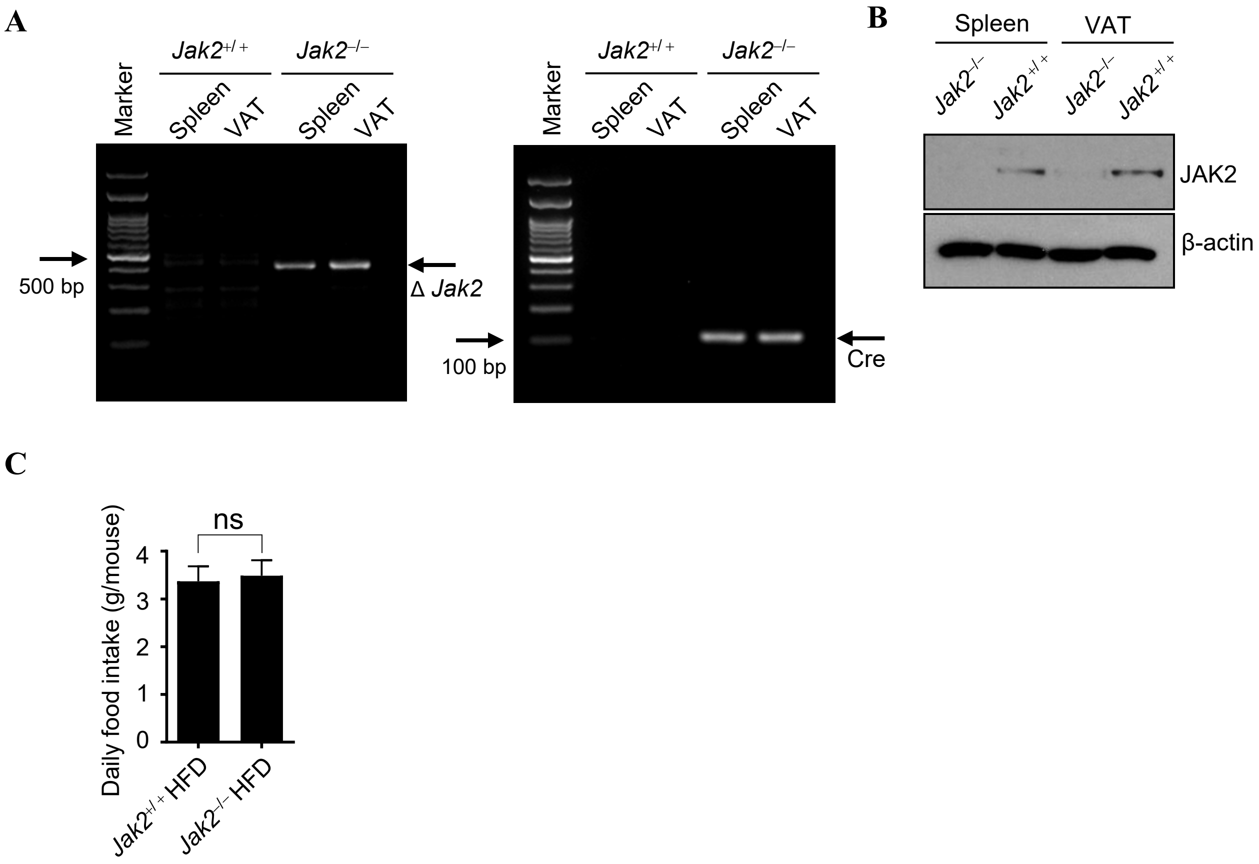

Supplemental Figure S2. *Jak2* ablation in CD11b$^+$ myeloid cells from *Jak2*$^{+/+}$ and *Jak2*$^{–/–}$ mice. (A) PCR analysis to measure Cre and Cre-induced deletion of *Jak2* (Δ*Jak2*) in genomic DNA isolated from CD11b$^+$ myeloid cells. Data are pooled from 3-4 mice. (B) Western blotting showing JAK2 protein expression in CD11b$^+$ myeloid cells from spleen and VAT. Date are pooled from 3-4 mice. (C) Food intake was monitored for 1 week in *Jak2*$^{+/+}$ and *Jak2*$^{–/–}$ mice on a HFD , n = 8; ns, not significant.

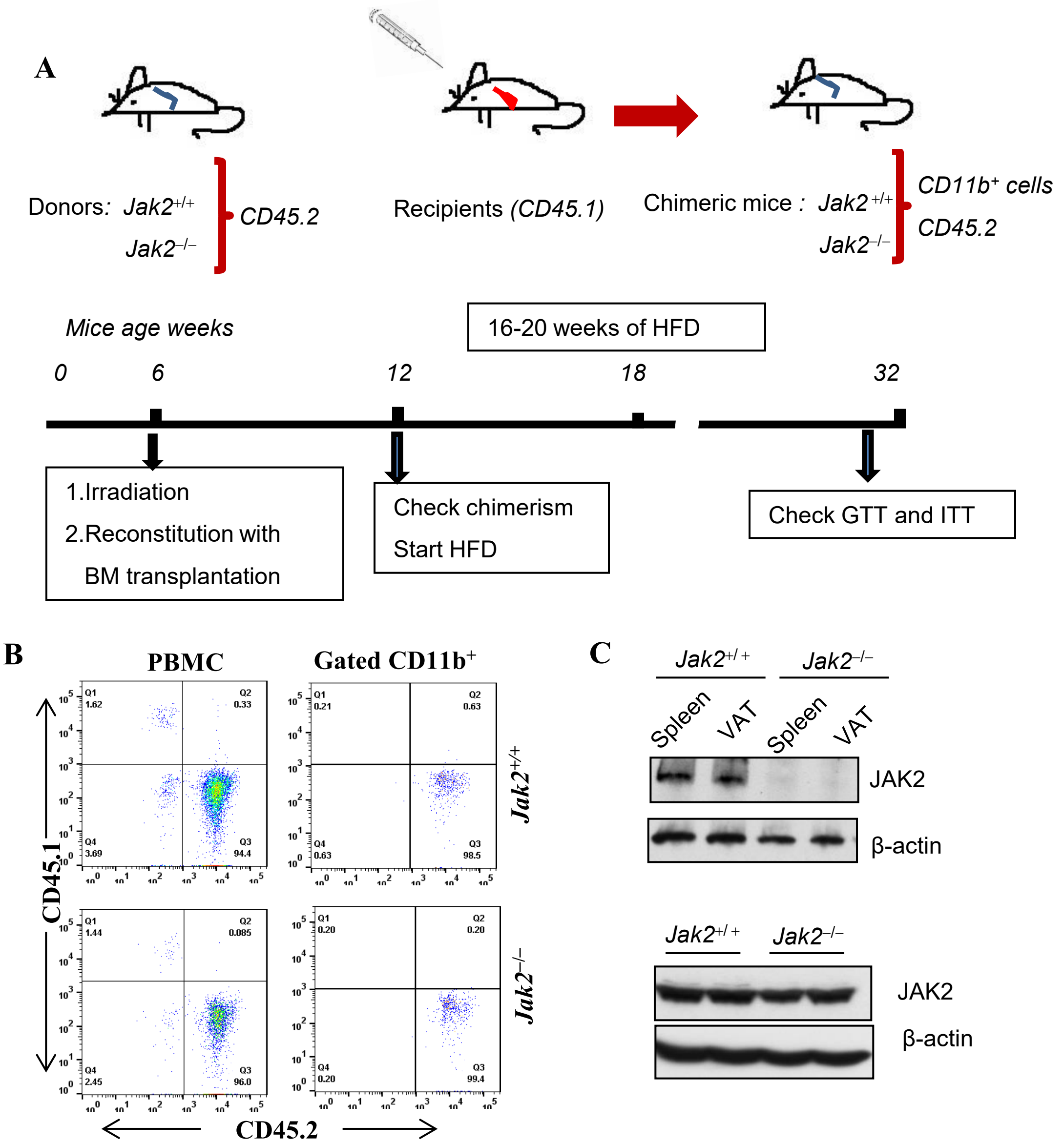

Supplemental Figure 3

Supplemental Figure S3. Generation of chimeric mice lacking *Jak2* in myeloid cells but not in hepatocytes. (A) Depiction of generating the chimeric mice used in this study (top). Scheme of experiment involving the chimeric mice (bottom). (B) Analysis of reconstitution efficiency for $CD11b^{+}$ myeloid cells. Flow cytometry analysis to measure CD45.1 (recipient) and CD45.2 (donor) expression on CD11b+ myeloid cells from the chimeras. (C) Western blotting showing JAK2 protein expression in $CD11b^{+}$ myeloid cells (top) and hepatocytes (bottom) from chimeric *Jak2*$^{+/+}$, *Jak2*$^{-/-}$ mice. Date are pooled from 3-4 chimeric *Jak2*$^{+/+}$ and *Jak2*$^{-/-}$ mice.

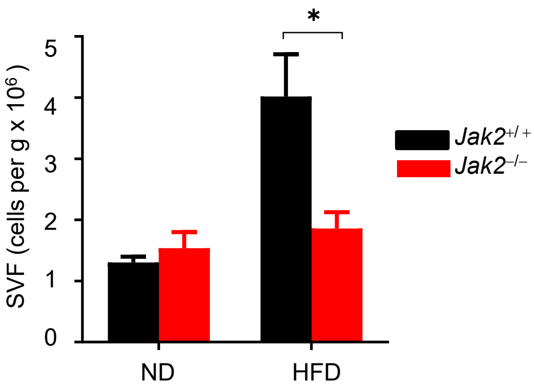

Supplemental Figure S4. *Jak2* in myeloid compartment promotes inflammation in diet-induced obesity mice. Absolute number of VAT-infiltrated cells from *Jak2*$^{+/+}$ and *Jak2*$^{-/-}$ mice on a ND or HFD; mean ± SEM, n = 10 mice, *p < 0.05.
